# Dynamics expression of *DmFKBP12/Calstabin* during embryonic early development of *Drosophila melanogaster*

**DOI:** 10.1186/s13578-019-0270-6

**Published:** 2019-01-08

**Authors:** Rui Feng, Xin Zhou, Wei Zhang, Tao Pu, Yuting Sun, Rong Yang, Dan Wang, Xiaofei Zhang, Yingfeng Gao, Zhenlu Cai, Yu Liang, Qiuxia Yu, Yajun Wu, Xinjuan Lei, Zhijia Liang, Odell Jones, Liyang Wang, Mengmeng Xu, Yanping Sun, William B. Isaacs, Jianjie Ma, Xuehong Xu

**Affiliations:** 1 National Engineering Laboratory for Resource Development of Endangered Crude Drugs in Northwest of China/CGDB, Shaanxi Normal University College of Life Sciences, Xi’an, 710062 China; 2 Ohio State University School of Medicine, Columbus, OH 43210 USA; 3 Beth Israel Deaconess Medical Center, Harvard Medical School, Boston, MA 02215 USA; 4 University of Pennsylvania ULAR, Philadelphia, PA 19144 USA; 5 Medical-Scientist Training Program, Department of Pharmacology, Duke University Medical Center, Durham, NC 27710 USA; 6 College of Pharmacy, Xi’an Medical University, Xi’an, 710062 China; 7 Johns Hopkins School of Medicine, Baltimore, MD 21287 USA

**Keywords:** *Drosophila* RyR-FKBP12, DmFKBP12 dynamic profile, Embryonic development

## Abstract

**Background:**

Calcium signaling are conserved from invertebrates to vertebrates and plays critical roles in many molecular mechanisms of embryogenesis and postnatal development. As a critical component of the signaling pathway, the RyR medicated calcium-induced calcium release signaling system, has been well studied along with their regulator FK506-binding protein 12 (FKBP12/Calstabin). Lack of FKBP12 is known to result in lethal cardiac dysfunction in mouse. However, precisely how FKBP12 is regulated and effects calcium signaling in *Drosophila melanogaster* remains largely unknown.

**Results:**

In this study, we identified both temporal and localization changes in expression of *DmFKBP12,* a translational and transcriptional regulator of *Drosophila* RyR (DmRyR) and FKBP12, through embryonic development. *DmFKBP12* is first expressed at the syncytial blastoderm stage and undergoes increased expression during the cellular blastoderm and early gastrulation stages. At late gastrulation, *DmFKBP12* expression begins to decline until it reaches homeostasis, which it then maintains throughout the rest of development. Throughout these described changes in expression, DmFKBP12 mRNA remain stable, which indicates that protein dynamics are attributed to regulation at the mRNA to protein translation level. In addition to temporal changes in expression, dynamic expression profiles during *Drosophila* development also revealed DmFKBP12 localization. Although DmFKBP12 is distributed evenly between the anterior to posterior poles of the blastoderm egg, the protein is expressed more strongly in the cortex of the early *Drosophila* gastrula with the highest concentration found in the basement membrane of the cellular blastoderm. Fertilized egg, through the profile as under-membrane cortex distribution concentering onto basement at cellular blastoderm, to the profile as three-gem layer localization in primitive neuronal and digestion architecture of early *Drosophila* gastrula. By late gastrulation, DmFKBP12 is no longer identified in the yolk or lumen of duct structures and has relocated to the future brain (suboesophageal and supraesophageal ganglions), ventral nervous system, and muscular system. Throughout these changes in distribution, in situ *DmFKBP12* mRNA monitoring detected equal distribution of *DmFKBP12* mRNA, once again indicating that regulation of *DmFKBP12* occurs at the translational level in *Drosophila* development.

**Conclusion:**

As a critical regulator of the DmRyR-FKBP complex, DmFKBP12 expression in *Drosophila* fluctuates temporally and geographically with the formation of organ systems. These finding indicate that *DmFKBP12* and RyR associated calcium signaling plays an essential role in the successful development of *Drosophila melanogaster*. Further study on the differences between mammalian RyR-FKBP12 and *Drosophila* DmRyR-FKBP12 can be exploited to develop safe pesticides.

**Electronic supplementary material:**

The online version of this article (10.1186/s13578-019-0270-6) contains supplementary material, which is available to authorized users.

## Introduction

In mammals, calcium signaling plays critical roles in many biological functions with its molecular mechanism in cell [1–3]. Abnormality of the signaling leads life-threatening diseases including cancers. Extracellular environmental homeostatic calcium is regulated through cell membrane integrated protein CaSR (calcium-sensing receptor) and ITG (integrins) [4, 5], and cytoplasmic calcium is controlled by inositol 1,4,5-trisphosphate receptors (IP3R) plus ryanodine receptors (RyR) in endoplasmic reticulum (ER) via one of well-known calcium induced calcium release (CICR) signaling pathway [6–9]. The CICR pathway as one of important calcium signaling pathways in cell functions through binding with their regulators such as FKBP12 (also known as Calstabin) and FKBP12.6 [10–15]. In insect, *Drosophila melanogaster* ryanodine receptor (*DmRyR*) cDNA was cloned from lava and the physical features of DmRyR single channel were characterized with in vitro overexpression system [16]. As a unique isoform of RyR in insect, the binding protein DmFKBP12 is essential as well in insect physiological and cellular processes. FKBP proteins are FK506 binding protein family implicating with many cellular function including calcium receptor signaling, protein folding and trafficking, transaction control, apoptotic death, and up to physiological role including embryonic development, stress response, tumorigenesis, neuronal response, angiogenesis and vascular remodeling [6, 7, 17].

Previous study unveiled that, in *Drosophila melanogaster*, the eight known DmFKBPs share homology with the *Homo sapiens* FKBP12 among its paralogues and orthologues through molecular phylogenetic analysis of FKBP family proteins [17]. Apart from DmFKBP59 possessing two FKBP domains, all other DmFKBPs including DmFKBP12, DmFKBP13, DmFKBP14, DmFKBP39, Shutdown (CG4736) and other two unnamed DmFKBPs (CG1847 and CG5482) compose of a single FKBP domain along with Cwf/Cwc superfamily domain, EF-band domain and TPR domain individually according to references from NCBI to FlyBase [17]. Because lack of *HmFKBP12* generating the newborn defects with the cerebral edema and cardiac arrhythmia [18], and *FKBP12* overexpression led to lethal defect of arrhythmic pathology [19], a critical function of DmFKBP12 is expected on *Drosophila* physiological role. Recently, the data from *DmFKBP12* mutant (also known as *Calstabin* and *FK506*-*BP2*) demonstrated that DmFKBP12 is necessary for S107 to play its critical role on extension of both health and life span against oxidative stress, and DmRyR is essential for larval development in *Drosophila* flies [20, 21].

FKBP12 and FKBP12.6 are widely recognized as the regulators of RyRs associated many calcium signaling physiological function [6–8, 17, 21]. RyR conducted calcium sparks regulated by FKBP12 which were first found in activation of Medaka fish eggs [22, 23] in zygote early development expressing critical function of FKBP12 [7]. To identify function of *DmFKBP12* in early *Drosophila* development, in this study, we dynamically localized the distribution pattern of DmFKBP12 protein and mRNA at different embryonic stage. The information obtained in this research provides more comprehension on how *DmFKBP12* performing its role within its dynamic distribution. Our data may benefit on developing more insect-specific pesticide targeting on early stage of insect embryo with more effective strategy related to DmRyR-FKBP12 complex.

## Materials and methods

### Fly stocks

All *Drosophila melanogaster* flies and embryos were grown at 25 °C on standard cornmeal-molasses-agar medium until collection and fixation. The wild-type embryos were from Canton S strain. Embryos were staged as previously described [24]. All animals maintained in natural day-night cycle. All animal maintenance and experiments were performed under guidelines approved by the Animal Care and Use Committee of Xi’an for Animal Use of Universities in Shaanxi Province, P.R. China.

### Embryo histological and morphological analysis

*Drosophila* embryos were collected at two hrs (syncytial blastoderm, 90.8%), three hr (cellular blastoderm, 91.5%), 12 h (early gastrula, 91.1%) and 24 h (late gastrula, 93.3%) after egg lay on grape juice agar plates (2.5% sucrose, 2.5% agar, 25% grape juice, 0.5% propionic acid), then dechorionated in 50% bleach and rinsed thoroughly in phosphate-buffered saline (PBS). Embryos were fixed in 50% heptane and 50% PEMFA (100 mM PIPES, 2.0 mM EGTA, 1.0 mM MgSO4, pH to 6.9 using KOH and 4% formaldehyde) for one hr and washed 3 times with PBS. Then samples were fixed in Bouin solution, paraffin embedded and sectioned at thickness of 8-10 μm [25]. Sections were and stained with hematoxylin-eosin (H&E) and periodic acid-silver methenamine (PASM) for regular histological analysis [26, 27].

### Immunohistochemistry

Deparaffinised and rehydrated sections were rinsed in PBS, and the antigens of samples were retrieved with preheated (approx. 100 °C) target retrieval solution (Dako, USA) for 20 min. Sections were treated with 0.3% H_2_O_2_ in methanol for 10 min and followed by a blocking step with 1% bovine serum albumin (BSA) diluted in PBS for 60 min at room temperature (RT). Then sections were incubated with primary antibody (anti-FKBP12 at 1:1000, sc-28814, Santa Cruz). After washed with PBS, the Envision-plus detection system was applied with HRP labelled polymer conjugated with secondary antibodies (anti-Rabbit, EnVision + System-HRP, Dako). Reaction products were visualized after incubation with 3, 3′-diaminobenzidine [28, 29].

The anti-FKBP12 antibody is a primary antibody designed against mouse FKBP12. And this antibody can also recognizes *Drosophila* FKBP12 because of that mouse FKBP12 and *Drosophila* FKBP12 shares 77% identity of total sequence of amino acids, 87% positive of the amino acid sequences composed of acidic side chain, basic side chain and uncharged polar chain with 0% gaps evaluated by compositional matrix adjustment [17]. A FKBP12.6 comparison of *Drosophila,* human and mouse show below with polar acids S T Y N Q and C in light-gray highlight, positive charged acids R K and H in medium-gray highlight, and negative charged acids D and E in dark-gray highlight.
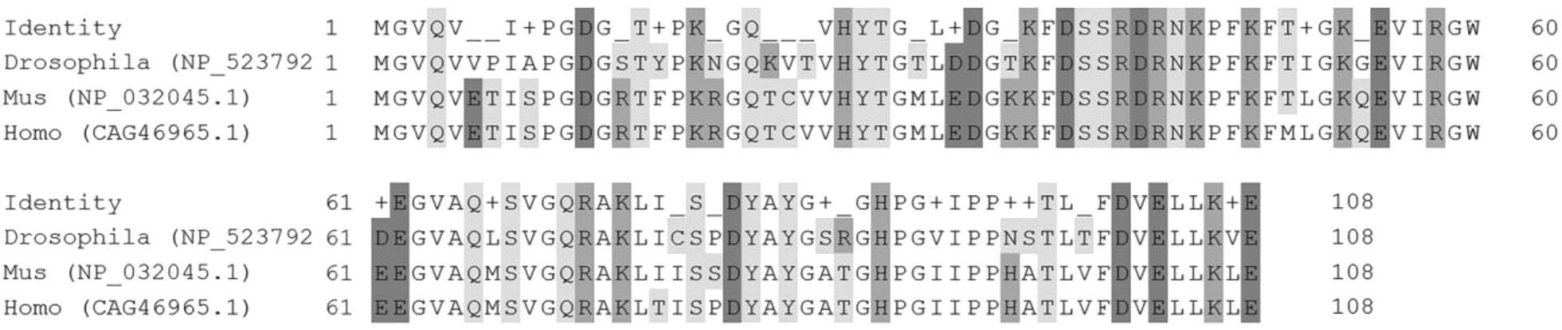


### Total RNA preparation and RT-PCR

Embryos were collected and snap frozen in liquid nitrogen. Total RNA was extracted with Trizol reagent (Invitrogen) and first-strand cDNA of DmFKBP12 was amplified from 3 µg of purified total RNA through reverse transcription polymerase chain reaction (RT-PCR) (Life Technologies) [9, 30]. Forward primer (Primer-F, 5′-CTAGCTAGCCGATGGGCGTACAAGTA GTTCCA-3′) and reverse primer (Primer-B, 5′-TACGAGCTCCTATTCGACCTTGAGCAGCTC-3′) were designed according to the published cDNA sequence of DmFKBP12 as *FK506*-*binding protein 2* of *Drosophila melanogaster* (NM_079068.5). Specific sequences for identifying restriction enzyme *Nhe* I and *Sac* I were included in the primer pairs at the 5′ and 3′ ends as restriction sites, respectively. The expression of *DmFKBP12* mRNA was assessed and the products were visualized with gel imaging system.

RT-PCR analysis was also used to identify *DmRyR* in developing embryos. Two fragments (I + II, and II + IV) of DmRyR domain (I + II, and II + IV) were amplified as their first-strand cDNA and then PCR fragments were cloned into pGME-T plasmid with forward primer-1 (5′-agatgtgggctctaaaca-3′) and reverse primer-1 (5′-tgaagatctcgttgggca-3′), forward primer-2 (5′-gagacatccgatccgata-3′) and reverse primer-2 (5′-cctcgttctggaattcgt-3′). The cDNA of DmRyR domain was sequenced for confirmation on their correctness (Additional file 1: Figure S1).

### RNA in situ hybridization

*Drosophila* embryo cDNA was amplified by PCR with two primers (5′-CTAGCTAGCCGCCACCATGGGCGTACAAGTAGTTCCA-3′, and 5′-TACGAGCTCCGCCACCTTC GACCTTGAGCAGCTC-3′), and then cloned into linearized pGEM-T Easy plasmid vector to make circular plasmid vector with the probe cDNA under down stream of SP6 and T7 promoters. The probes were synthesized for RNA labeling with digoxigenin-UTP by in vitro transcription with SP6 and T7 RNA polymerase (#11175025910, Roche). Antisense double digoxigenin (DIG) labeled RNA was used as a probe of Dm*FKBP12* hybridization, and DIG labeled sense RNA was used as a negative control (data not shown). The probes were quantified and applied 1 μg of probe per hybridization reaction. Probes were detected using a primary sheep anti-DIG-AP 1:10,000 (#11093274910, Roche) and NBT/BCIP for the sensitive detection [31].

All *Drosophila* embryo sections collected at syncytial blastoderm, cellular blastoderm, early and late gastrulation stages, were prepared according to the previous description above with 0.01% DEPC pre-treated water [31]. DEPC overnight treatment was applied on all accessories used for the in situ hybridization.

### Western blotting analysis

Expression analysis on the DmFKBP12 protein of *Drosophila* embryos was inspected by Western blotting according to the standard protocol [9, 32]. Tissue proteins from embryos was extracted using RIPA reagent. Standard BSA approach was used for protein quantification. The proteins sample were denatured after heated at 100 °C and separated on a gradient SDS-PAGE, and then transferred onto polyvinylidene difluoride (PVDF) membranes with a BioRad transfer unit at 100 V for 120 min. The membranes were blocked with 5% non-fat milk in TBST at 4 °C overnight and detected with primary antibody (anti-β-tubulin 1:5000; anti-FKBP12 1:1000, sc-28814, Santa Cruz) at room temperature (RT). After three times washing with TBST, the membranes were incubated with secondary antibody at RT. Immuno-detection was carried out with ECL followed by exposure to motored molecular imaging system (Tanon 4200, China). Images were quantitated using the Image J software downloaded from website of National Institutes of Health [16].

### Microscopy and statistical analysis

The histochemistry images were documented and analyzed with the inverted microscopy (Carl Zeiss Microscopy GmbH) and manufacturer software ZEN. Data were presented as mean ± SEM, and were analyzed with SPSS 22.0 and Graphpad Prism 7. A one-way ANOVA followed by a post hoc comparison Tukey was employed to analyze the data. A *P* value less than 0.05 and 0.01 was considered statistically significant [16, 27].

## Results

### DmFKBP12 during *Drosophila* embryogenesis

The *Drosophila* embryonic samples were collected at four different stages i.e. syncytial blastoderm, cellular blastoderm, early and late gastrulation stages according to the approach described in the previous publication [33]. These four-stage *Drosophila* embryos harvested and sorted at following four timing-points for inspection of DmFKBP12 expression during *Drosophila* development. The embryos from syncytial blastoderm stage were sorted from collection of 2 h after fertilization (syncytial blastoderm, 90.8%; n > 400). The embryos of cellular blastoderm stage (cellular blastoderm, 91.5%; n > 300), early gastrulation stage (early gastrula, 91.1%; n > 200) and late gastrulation stage (late gastrula, 93.3%; n > 200) were done individually from 3 h, 13 h and 24 h collection after fertilization.

**Fig. 1:**
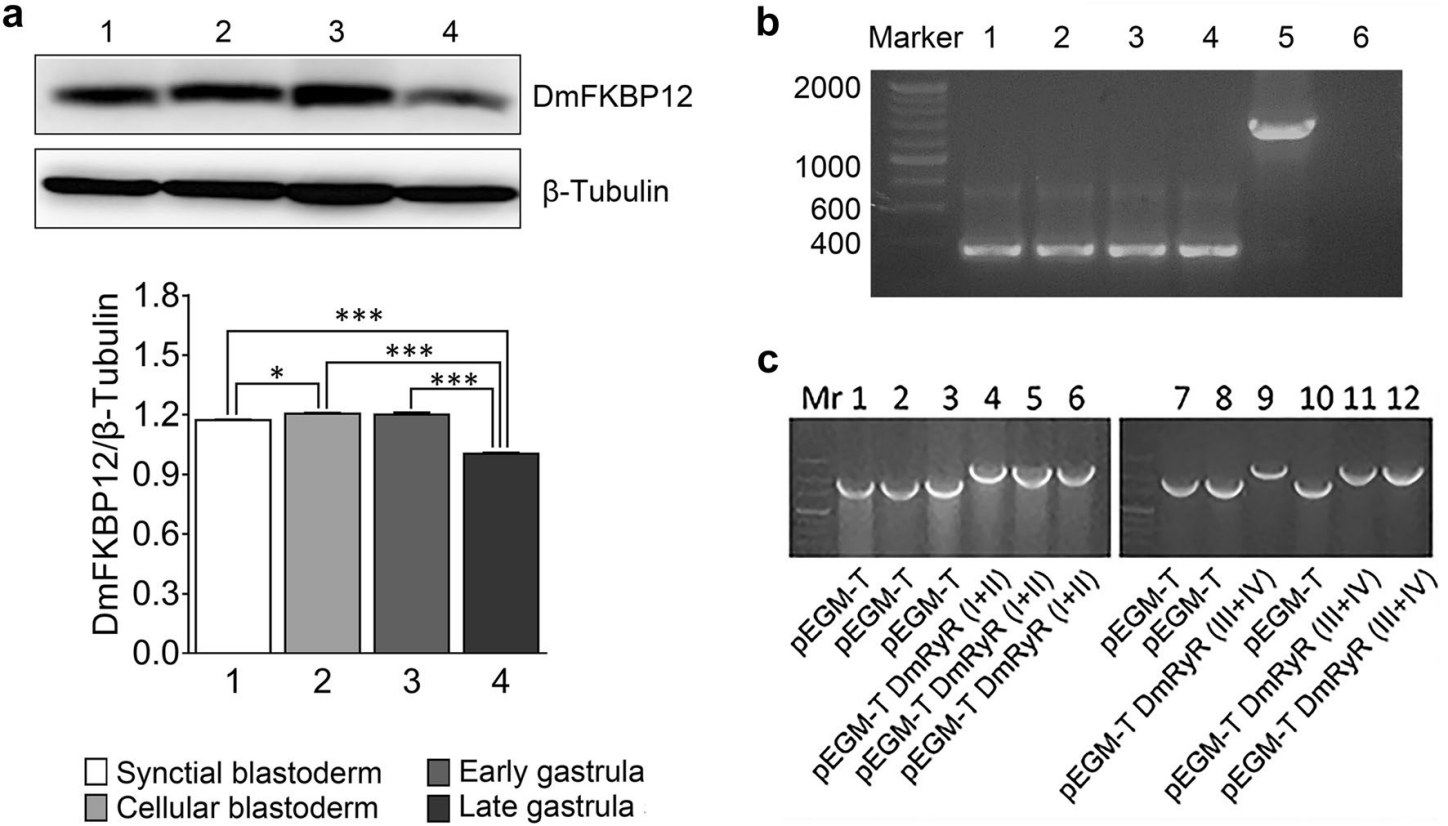
The expression patterns of DmFKBP12 protein and its mRNA during *Drosophila* embryogenesis within four stages syncytial blastoderm, cellular blastoderm, early and late gastrulation. **a** Expression of DmFKBP12 protein by Western blot at the four stages during *Drosophila* embryonic development. **b** Displays RT-PCR analysis of the expression of *DmFKBP12* mRNA during different embryonic developmental stages. (1) Syncytial blastoderm; (2) Cellular blastoderm; (3) Early gastrulation stage; (4) Late gastrulation stage; (5) Positive control; (6) Negative control. β-Tubulin served as internal control. Data are expressed as mean ± SEM. *p < 0.05, **p < 0.01 and ***p < 0.001. The two fragments of DmRyR domain were sub-cloned into pGME-T easy examined as **c**. The digestion pattern of restriction enzymes exhibited of clones with the cDNA DmRyR domain (I + II) (**c**-4, -5 and -6) compared to blank vector (**c**-1, -2 and -3). For the cDNA DmRyR domain (III + IV), the digestion pattern of clones showed as the plasmids with cDNA DmRyR domain (III + IV) (**c**-9, -11 and -12) compared to blank vector (**c**-7, -8 and -10)

The DmFKBP12 expression were detected within the *Drosophila* embryo lysis with Western blotting analysis in four stages (Fig. 1a). Normalized to the β-tubulin protein, the expression initiated at stage of syncytial blastoderm, increased at stage of cellular blastoderm (lane 1 and 2; *p *< *0.05*) and kept constant at stage of early gastrula (*p *> *0.05*) until stage of late gastrula (lane 3 and 4). In the late stage of gastrula embryos (lane 4), the DmFKBP12 expression dramatically decreased compared to syncytial and cellular blastoderm (both *p *< *0.001*). Meanwhile, during the *Drosophila* gastrulation, the protein expression of DmFKBP12 in the late stage are dramatically decreased and kept at detectable level (lane 3 and 4; *p *< *0.001*).

RT-PCR analysis of *DmFKBP12* gene expression supported the result of protein analysis (Fig. 1b). In the total RNA samples of syncytial blastoderm, cellular blastoderm, early and late gastrulation stages, the *DmFKBP12* cDNA fragment amplified from transcripted first strain cDNA were clearly detected in four-stage *Drosophila* embryos. The above inspection demonstrated that the *Drosophila DmFKBP12* expression was examied at both translation and transcription levels. Our data extend the detected expression of *DmFKBP12* from adult *Drosophila* [20] to very early embryonic syncytial blastoderm stage of its development with multiple nuclei of first 13 mitosis after fertilization [34].

As we know that *DmFKBP12* is the regulator of *DmRyR*, the major component of Calcium signaling on CICR pathway, we further examined its existence within the *Drosophila* embryos by cloning its most conserved RyR domain of all RyR orthologues. The cDNA sequencing confirmation provided the evidence on the appearance of *DmRyR* in *Drosophila* embryos (Additional file 1: Figure S1).

### Dynamic profile of DmFKBP12 protein from syncytial blastoderm to cellular blastoderm of the *Drosophila* embryo

**Fig. 2:**
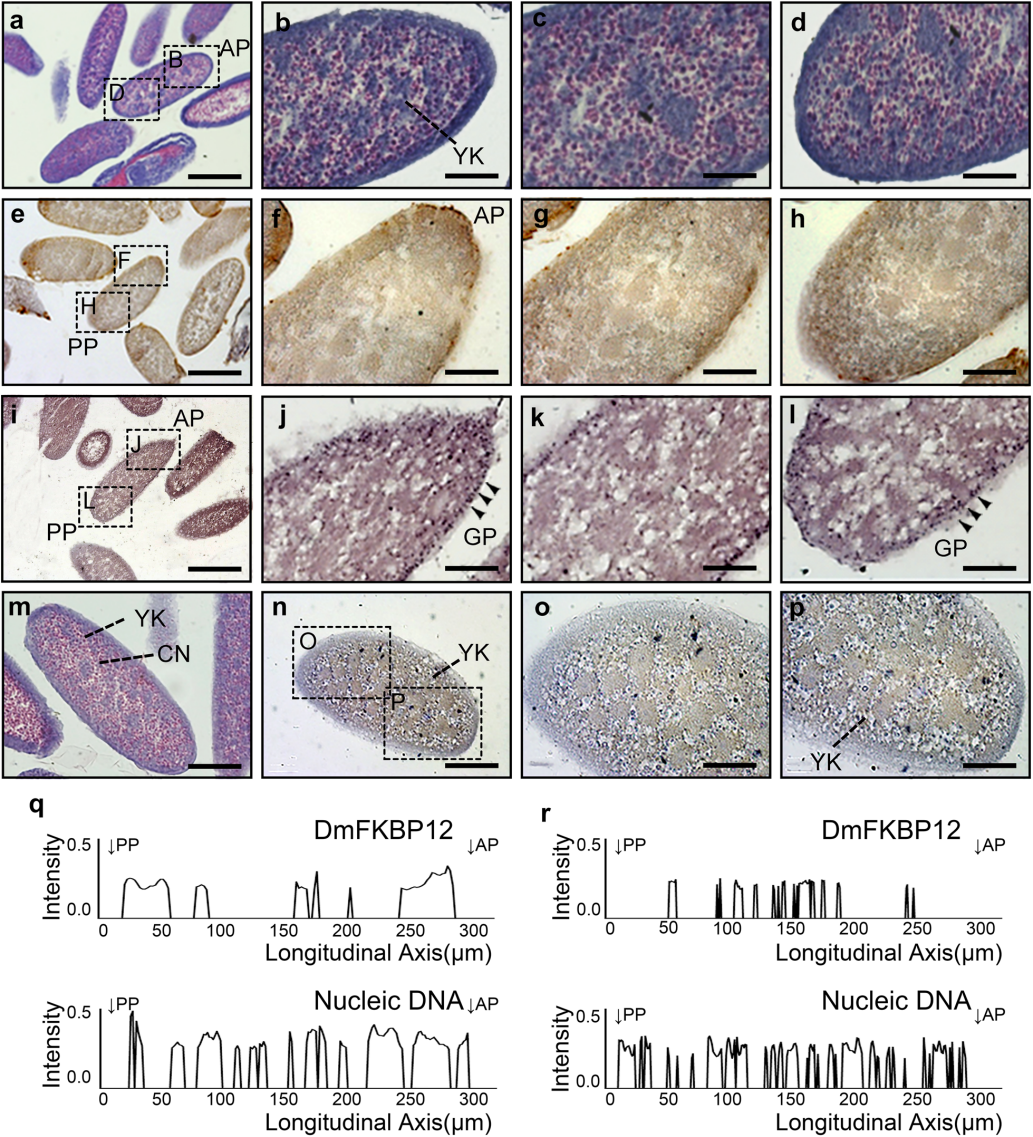
Expression profile of DmFKBP12 protein in syncytial blastoderm of the *Drosophila* embryo. **a**–**d** H&E staining of the syncytial blastoderm. This stage of *Drosophila melanogaster* embryogenesis implied 13 rapid nuclear divisions within a common cytoplasm. And these nuclear divisions produced roughly 300–400 nuclei by the end of the ninth division. **e**–**h** and **n**–**p** display the distribution of DmFKBP12 protein in the syncytial blastoderm. The DmFKBP12 was expressed in embryonic cytoplasm and plasma membrane, restricted to the periphery, anterior and posterior of the embryo before cellularization. **i**–**l** show PASM staining of syncytial blastoderm. Glycoprotein granules distribute in the periphery of the embryo. **m**–**p** presents early syncytial blastoderm in which nuclei are under division. The qualifications of DmFKBP12 distribution in early and late syncytial blastoderm are presented in **q** and **r**. Scale bar for **a**, **e** and **i** is 160 µm, for **m**, **n** is 80 µm, for **b**–**d**, **f**–**h**, **j**–**l** and **o**–**p** is 40 µm. AP, anterior pole of egg; PP, posterior pole of egg; YK, yolk; CN, cleavage nucleus; GP, glycoprotein

*Drosophila* syncytial blastoderm is formed from zygote after 13 mitosis of two haploid pro-nuclear meeting [34]. After fertilization, the diploid nuclear DNA synthesis and rapid nuclear divisions do not generate daughter cells but daughter nuclei without accomplishment of the plasma cytokinesis, and lead to syncytial blastoderm in a multinuclear cell [34]. At this stage, the multinuclear blastoderm cell was equipped with evenly distributed yolk particles (Fig. 2a–d) and protein granules (Fig. 2i–l) composing of glycoproteins within the blastodermal cytoplasm. The DmFKBP12 protein can be detected in entire embryo but with less differentiated localization (Fig. 2e–h, and q). The DmFKBP12 mainly localized in zones of both anterior pole (AP) and posterior pole (PP) of egg indicated with its density (Fig. 2q). The DmFKBP12 zone in posterior pole (Fig. 2h) is larger than the anterior pole (Fig. 2f) with weaker expression in middle zone of the egg (Fig. 2g, q). It is clear that the DmFKBP12 in both PP and AP zone are partially co-localized with glycoprotein granules. At the syncytial blastoderm stage, the single-cell *Drosophila* blastoderm with nuclear accumulation after 13 nucleic mitotic divisions are ready for cellularization process.

**Fig. 3:**
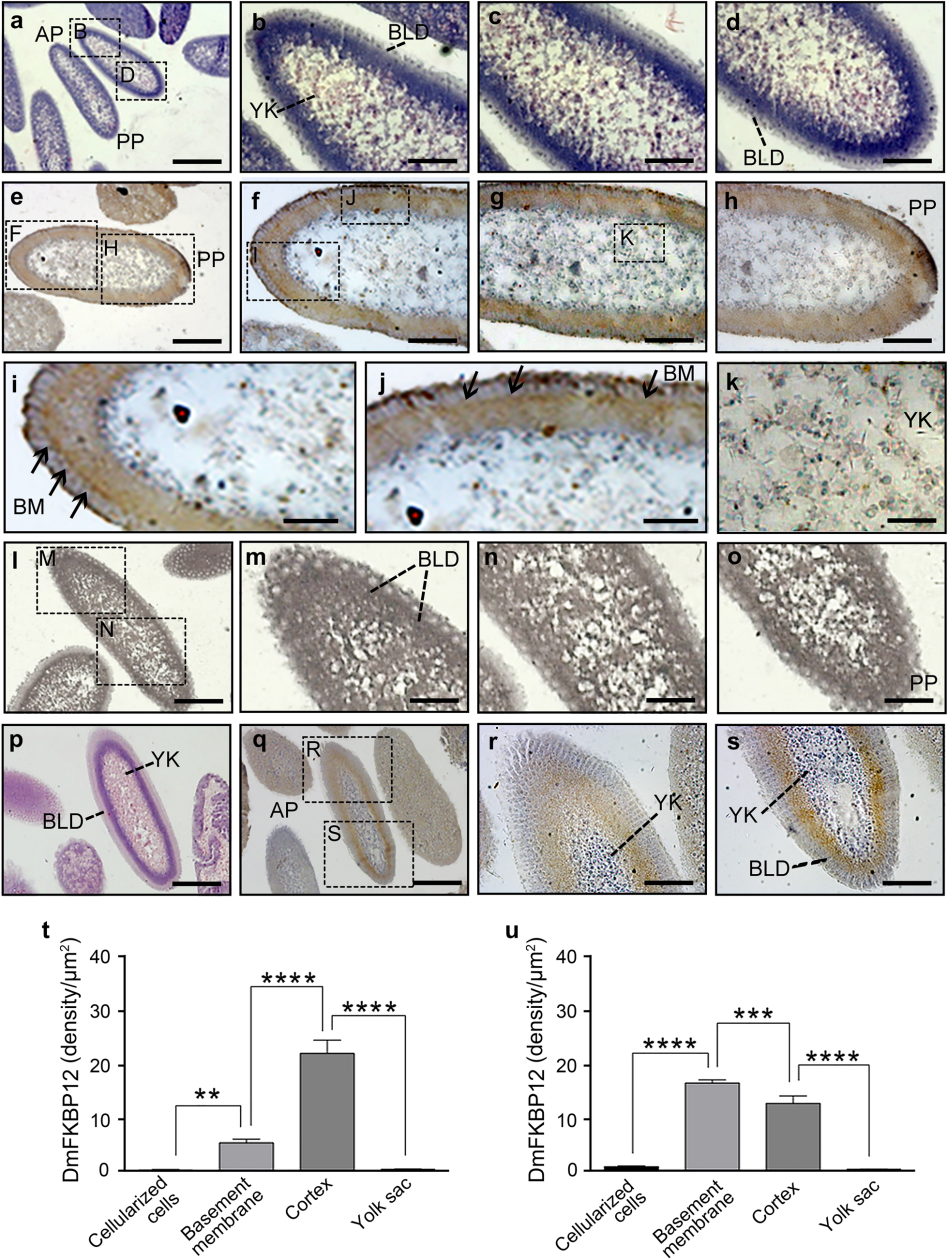
Expression profile of DmFKBP12 protein in cellular blastoderm of the *Drosophila* embryo. **a**–**d** H&E staining of the cellular blastoderm. The divided nuclei have reached the periphery and formed blastoderm. **e**–**h** and **q**–**s** exhibit the distribution of DmFKBP12 protein in the cellular blastoderm. **i**–**k** show the larger views of **f**–**g**. DmFKBP12 was mainly expressed in the periphery of the embryo. **l**–**o** Display PASM staining of cellular blastoderm. Glycoprotein granules increased at the cellular blastoderm stages and there were no obvious glycoprotein granules. **p**–**s** present earlier cellular blastoderm in **e**–**k**. The qualifications of DmFKBP12 distribution in late and early cellular blastoderm are presented in **t**, **u**. Scale bar for **a** is 160 µm, for **e**, **l** and **p**, **q** is 80 µm, for **b**–**d**, **f**–**h**, **m**–**o** and **r**–**s** is 40 µm, for **I**–**k** is 20 µm. BLD, blastoderm, nuclei and cell; YK, yolk; AP, anterior pole of egg; PP, posterior pole of egg; BM, Basement membrane. Data are expressed as mean ± SEM. *p < 0.05, **p < 0.01, ***p < 0.001 and ***p < 0.0001

While *Drosophila* syncytial blastoderm develops to cellular blastoderm, the four thousands nuclei generated after 13 mitosis migrate to subsurface of the single-cell blastoderm egg (Fig. 3a–d) and form embryonic trophectoderm lineage, with central yolk sac zone fussed from spread small yolk portions. The lineage constructs embryo as classic blastodermal subsurface-linearized single nucleus-cell sheet enveloping the surface of embryo. At this stage, the differentiated distribution of the DmFKBP12 protein continuously exhibited in the *Drosophila* blastoderm. The positive staining shifted to cortex zone apposition next to trophectoderm primitive epithelium (Fig. 3e–j, and u) with non-detectable staining in central yolk sac localization (Fig. 3e–h, k) along with migration of glycoprotein granules (Fig. 3l–o). With the development of the embryo, the DmFKBP12 proteins concentrated to basal-cell layer zone and aggregated in basement localization of blastodermal epithelial cells (Fig. 3q–s, t), where is critical tissue architectural structure for directing embryo further differentiation. The protein density in basement membrane is dramatically higher than that in the cellularized cell layer (*p *< *0.0001*) and significantly higher than that in the cortex zone as well (*p *< *0.001*).

### Dynamic profile of DmFKBP12 protein from early to late gastrulation stages of the *Drosophila* embryo

**Fig. 4:**
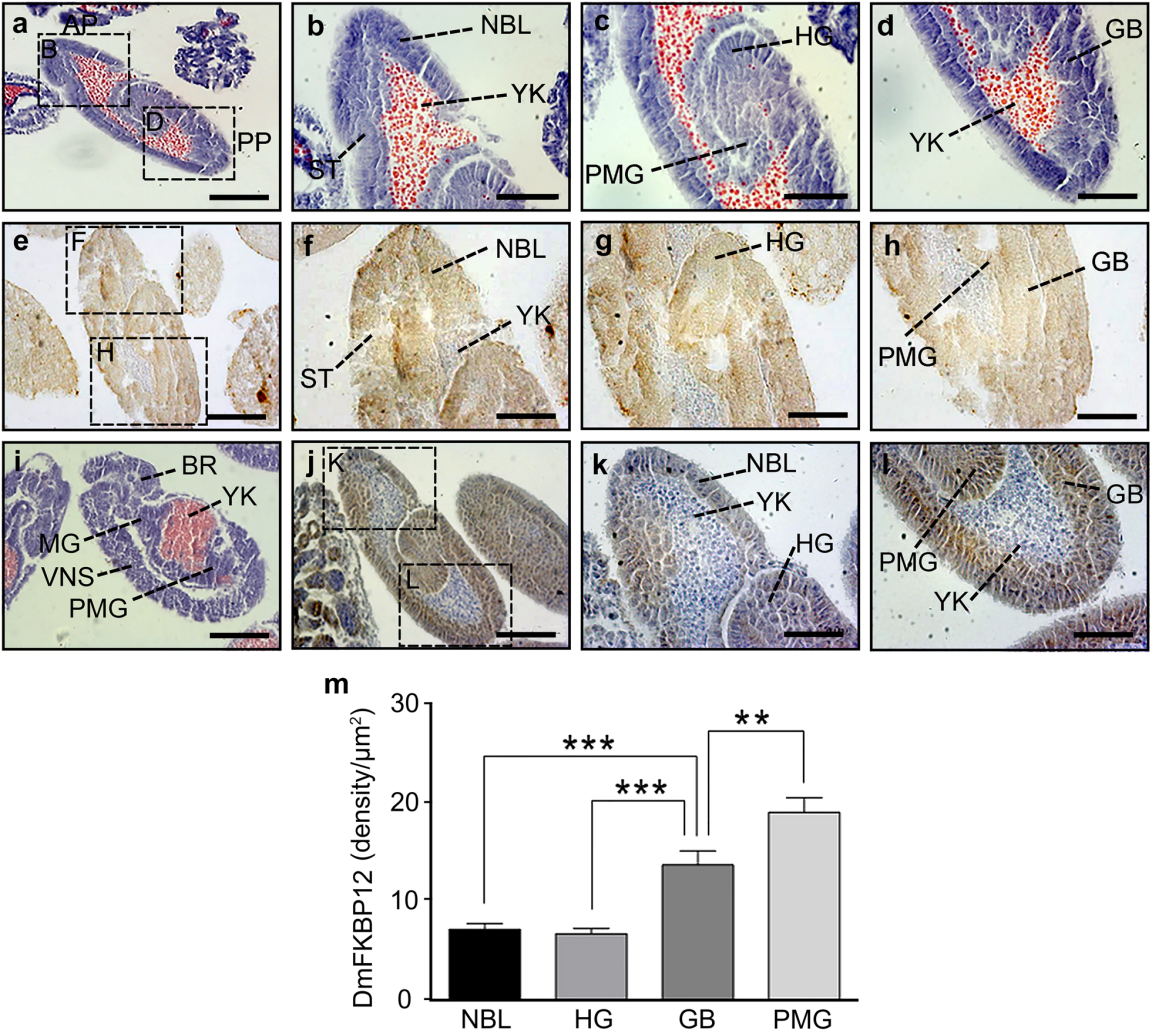
Expression profile of DmFKBP12 protein at early gastrulation stage of the *Drosophila* embryo. **a**–**d** and **i** H&E staining of embryo at early gastrulation stage. Three germ layers are developed and the mouth of the posterior invagination was forced anteriorly. **e**–**h** and **j**–**l** display the distribution of DmFKBP12 protein at early gastrulation stage. DmFKBP12 was mainly expressed in the developing gut, muscle and neuroblasts. **i**–**l** present earlier gastrulae in **e**–**h**. The qualifications of DmFKBP12 distribution in early gastrulae are presented in **m**. Scale bar for **a**, **e** and **i**–**j** is 80 µm, for **b**–**d**, **f**–**h** and **k**–**l** is 40 µm. AP, anterior pole of egg; PP, posterior pole of egg; NBL, neuroblasts; ST, stomodaeum; YK, yolk; HG, hindgut; PMG, posterior midgut rudiment; GB, germ band; BR, brain, supraesophageal ganglion; MG, midgut; VNS, ventral nervous system. Data are expressed as mean ± SEM. *p < 0.05, **p < 0.01 and ***p < 0.001

Dramatic differentiation in *Drosophila* embryo is promoted after accomplishment of cellular blastoderm in which “independent” cells after accomplishment of mitotic cytokinesis are configured from syncytial embryonic cells. In order to develop forwards to late *Drosophila* gastrula, the cellularized single-nuclear embryonic blastoderm cells began their migration continuously and arrived at their different location in early gastrulation stage. In the early stage, the cells preformed their preliminary folding to form primary three-germ layers. The mouth of the posterior invagination was forced from anteriorly portion of embryo (Fig. 4a–d). The *Drosophila* DmFKBP12 were involved into all germ layer by locating in newly formed cells from the basement localization at the cellular blastoderm stage, and distributing in neuroblasts (NBL) and stomodaeum (ST) but not in yolk (YK) within zone of anterior pole, and hindgut (HG), germ band (GB), and posterior midgut rudiment (PMG) within zone of anterior pole of embryo (Fig. 4e–h, and m). At the late-early gastrulation stage, distributing trend of *Drosophila* DmFKBP12 appeared towards to brain, supraesophageal ganglion (SG), ventral nervous system (VNS), midgut (MG), and posterior midgut rudiment (PMG) but clearly not in yolk (Fig. 4i–l, and m) with the equal densities in neuroblast and hindgut, higher expressions in germ band and posterior midgut rudiment compared to NBL and HG (Fig. 4m, all *p *< *0.01*).

As the fundamental events of early differentiation, association of cell proliferation and migration are tightly relevant with cellular calcium signaling as discussed previously. It is noticeable that DmFKBP12 is essential within stage transition from early gastrula to late gastrula.

**Fig. 5:**
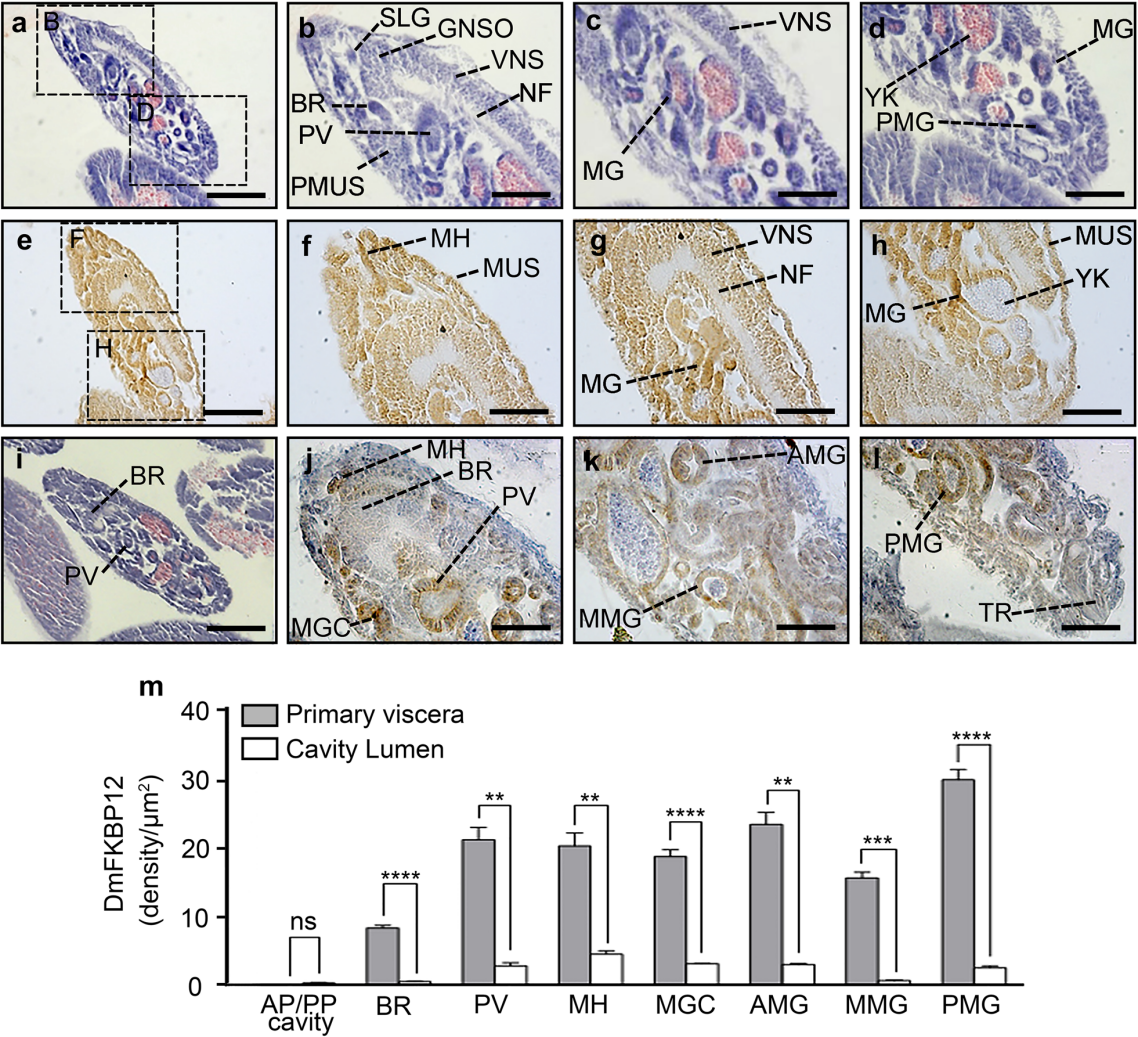
Expression profile of DmFKBP12 protein at late gastrulation stage of the *Drosophila* embryo. **a**–**d** and **i** H&E staining of embryo at late gastrulation stage. A number of different morphogenic movements has been completed. **e**–**h** and **j**–**l** display the distribution of DmFKBP12 protein at late gastrulation stage. DmFKBP12 was detected in the differentiated organs which involved in digestive, nervous and muscular system, such as mouth hook, midgut, middle midgut, midgut caecum. Orientation of the embryo is anterior to the top and posterior to the bottom. **i**–**l** present advanced late gastrulae than that in **e**–**h**. The qualifications of DmFKBP12 distribution in early gastrulae are presented in **m**. Scale bar for **a**, **e** and **i** is 80 µm, for **b**–**d**, **f**–**h** and **j**–**l** is 40 µm. MH, mouth hook; SLG, salivary gland; GNSO, suboesophageal ganglion; BR, brain, supraesophageal ganglion; AMG, anterior midgut rudipighian; PMUS, pharyngeal musculature; PV, proventriculus; MG, midgut; VNS, ventral nervous system; NF, nerve fibers and tracts; MUS, muscle; YK, yolk; ST, stomodaeum; PMG, Posterior midgut rudiment; MGC, midgut caecum; MMG, middle midgut; TR, trachea. Data are expressed as mean ± SEM. *p < 0.05, **p < 0.01, ***p < 0.001 and ***p < 0.0001

In late gastrulation stage, *Drosophila* embryo had relatively more distinguished complexity than that in the early and late-early stages on furthermore differentiation. Along with three-germ layers developing in gastrula, the detected expression of DmFKBP12 protein in the architecture of embryonic tissues and organs is getting more specific and restricted in certain location (Fig. 5). The expression was continuously detected in digestive, nervous and muscular system including mouth hook (MH), head muscle (MUC), pharyngeal musculature (PMUS), brain supraesophageal ganglion (BR), suboesophageal ganglion (GNSO), ventral nervous system (VNS), proventriculus (PV), midgut (MG), and salivary gland (SV) within zone of anterior pole and main body of embryo (Fig. 5a–h).

In *Drosophila* late gastrulation stage, a major recognizable difference from previous stages is that the non-distribution areas of the DmFKBP12 protein are much more noticeable beside yolk-zone surrounding central portion of nerve fibers and tracts (NF), luminal portion of the most tubular structures in entire body cavity (Fig. 5e–h). At the end of late gastrulation stage, the non-expression zone of DmFKBP12 protein is getting more and more discernible. Furthermore, inner germ layer differentiated organs/tissues expressing less DmFKBP12 are extremely eminent (Fig. 5i–l). Compared to our inspection on DmFKBP12 expression pattern in previous three stages of *Drosophila* development listed above, the protein distribution in the late gastrula is significant less than that in early gastrulation and other two stages according to our immunological detection. The DmFKBP12 protein expression distributed more in the cell-composed primary viscera including mouth hook, brain (supraesophageal ganglion), midgut caecum, proventriculus, anterior midgut rudipighian, middle midgut and posterior midgut rudiment, less located in their lumens (Fig. 5m, all *p *< 0.001). Weak signal of DmFKBP12 expressed in cavity of posterior and anterior poles can be detected.

### Dynamic mRNA profile of *DmFKBP12* gene expression of *Drosophila* embryogenesis from syncytial blastoderm via cellular blastoderm to gastrulation

As our dynamic data on expression of *DmFKBP12* in *Drosophila* embryogenesis, the distribution pattern of DmFKBP12 protein developed from syncytial blastoderm, via cellular blastoderm, to early and late gastrulation stages along with growing construction of primitive tissues/organs from three-germ layers. All differentiation from a single-cell embryo, via syncytial thousand-nuclei cell embryo, to classic single-nuclear cell embryo after cellularization, the *Drosophila* development accompanying with cellular DmFKBP12 is critical on morphological architecture. In data of this session, we focus on investigation of *DmFKBP12* gene expression on mRNA level to confirm the function of evolutional conceived *DmFKBP12* gene in *Drosophila* embryogenesis.

**Fig. 6:**
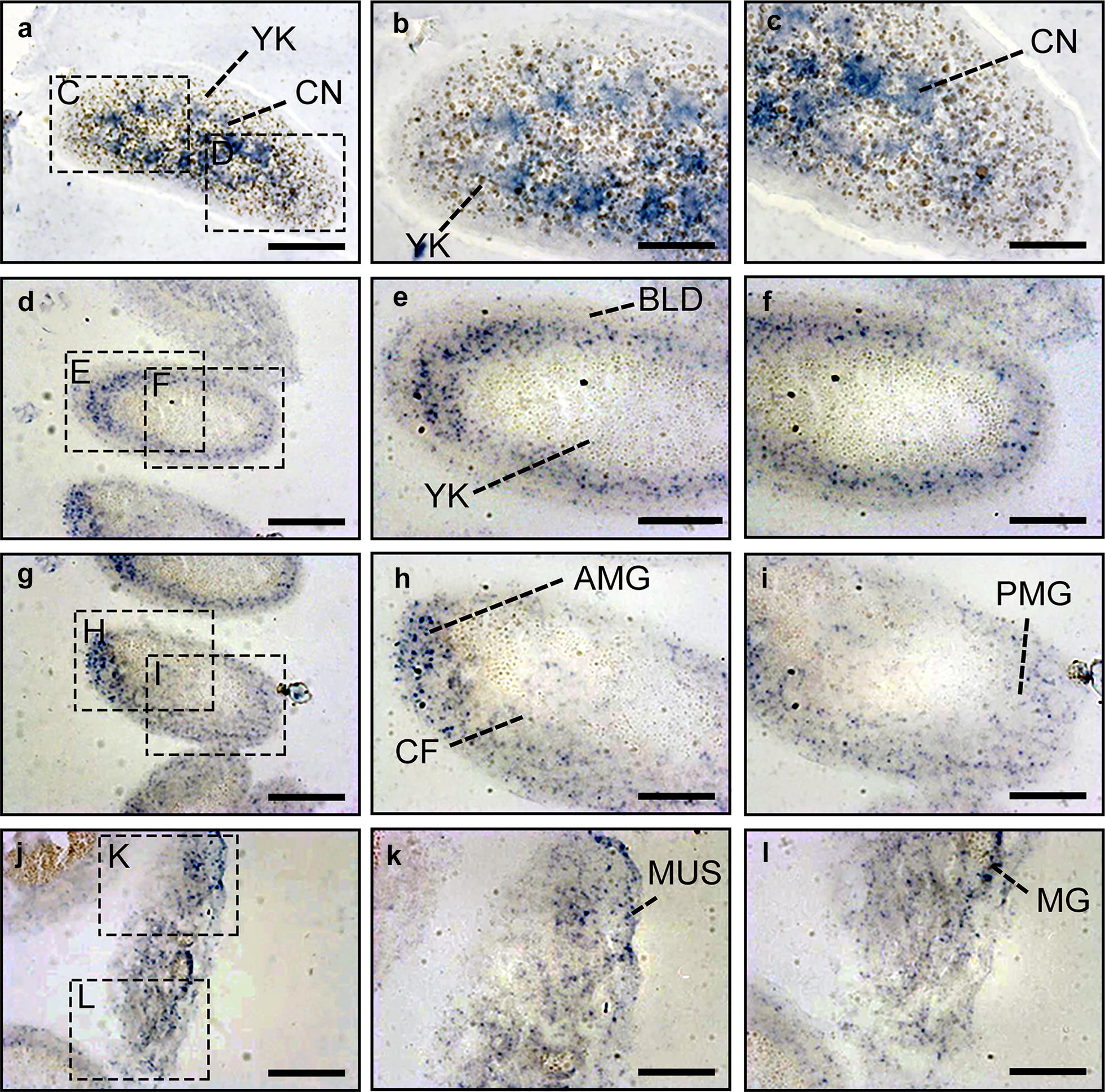
*In situ* localization of *DmFKBP12* mRNA during embryonic development of the *Drosophila* embryo. **a**–**c** show in situ localization of *DmFKBP12* mRNA at syncytial blastoderm stage. *FKBP12* mRNA was expressed in cytoplasm of the embryo. **d**–**f** display in situ localization of *FKBP12* mRNA at cellular blastoderm. FKBP12 mRNA located in the inner limits of blastoderm cells. The expression is higher in the anterior region of the embryo than that in the rear. **g**–**i** exhibit in situ localization of *FKBP12* mRNA at early gastrulation stage. It is mainly localized in the developing gut. **j**–**l** In situ localization of *FKBP12* mRNA at late gastrulation stage. The mRNA expressed in the muscle and gut. Scale bar for **a**, **d**, **g** and J is 80 µm, for **b**–**c**, **e**–**f**, **h**–**i** and **k**–**l** is 40 µm. YK, yolk; CN, cleavage nucleus; BLD, blastoderm, nuclei and cells; CF, anterior oblique cleft, cephalic furrow; AMG, anterior midgut rudipighian; PMG, posterior midgut rudiment; MUS, muscle; MG, midgut; AP, anterior pole of egg; PP, posterior pole of egg

We utilized RNA in situ hybridization analysis to dynamically inspect the mRNA of DmFKBP12 transcript from the *Drosophila* embryos harvested from four stages of syncytial blastoderm, cellular blastoderm and gastrulation including early and late gastrula (Fig. 6a–l). The *DmFKBP12* mRNA were evenly distributed within entire syncytial blastodermal embryo and left yolk zone free of the detected signal with multiple-small size stainless spots (Fig. 6a–c). In cellular blastodermal embryo, the mRNA were shifted towards embryo edge zone to subspace underneath cell membrane along with the moving of the thousands of nuclei, and eventually form the syncytial cell cortex zone and the central yolk zone. The massive *DmFKBP12* mRNA staining distributed in the cortex zone but not in the central zone (Fig. 6d–f). In the early gastrulation stage, three-germ layers started to be produced by aggravating towards inside of the embryo and push-shrink the DmFKBP12 mRNA free from the central yolk zone, the mRNA along with differentiated primitive germ layers were folded deeply towards into embryo to perform further cellular differentiation (Fig. 6g–i). While the *Drosophila* embryos development reached to late gastrula, three-germ layers already constructed more complicated tissues/organs with tracks and ducts of ventral nervous system and mouth hook, and luminal spaces of mouth hook, salivary gland, proventriculus and midgut rudiment. The DmFKBP12 mRNA staining always settled on solid portions of the tissues/organs including seating on primitive intra-epithelium of the structures and not within inner space of the primitive duct-tissues (Fig. 6j–l). Distribution of the *DmFKBP12* mRNA staining signals appeared positively change with the condensed cells indicated by cell nuclei staining.

**Fig. 7:**
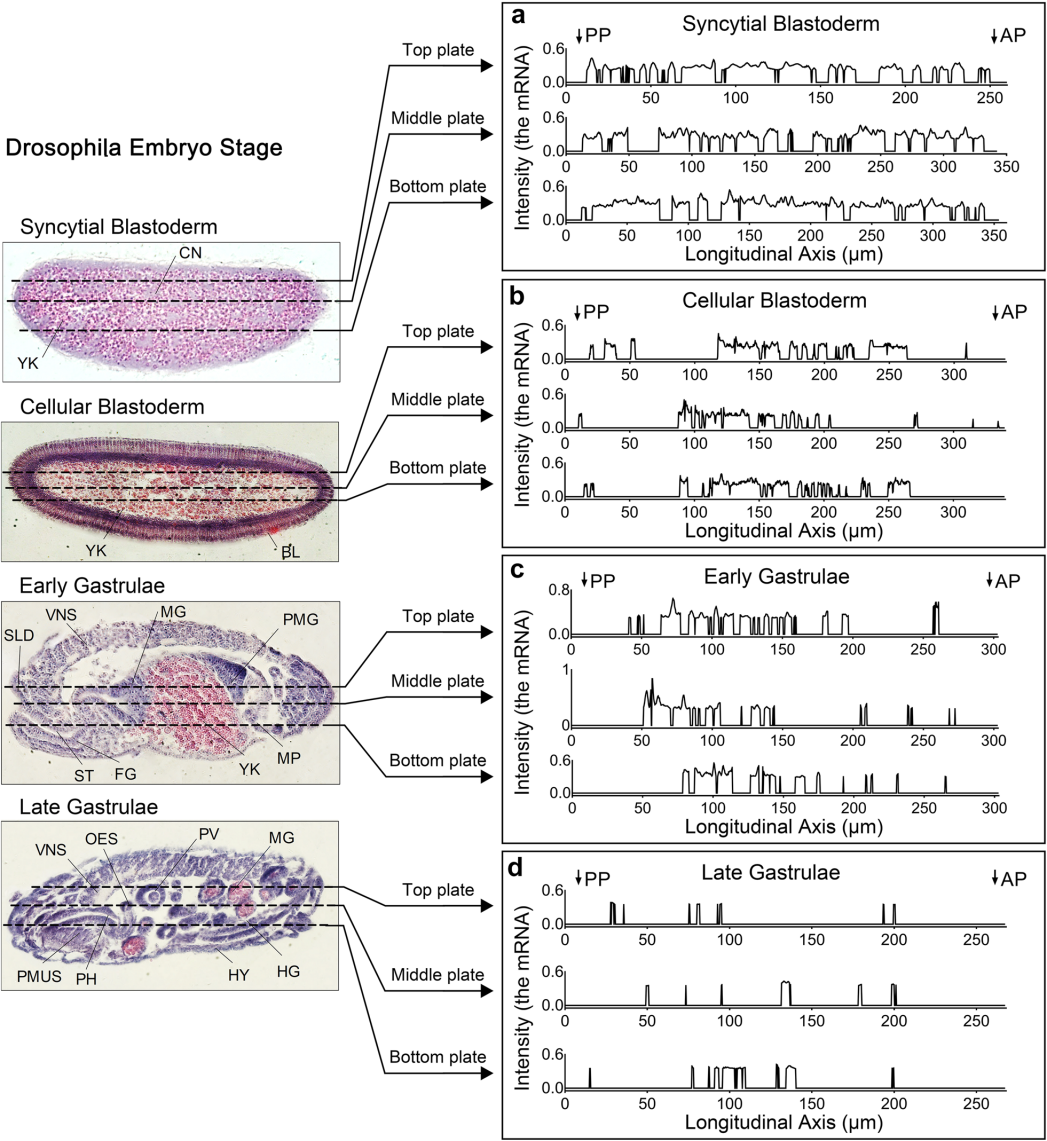
The schematic distribution of *DmFKBP12* mRNA during *Drosophila* embryogenesis within four stages of syncytial blastoderm, cellular blastoderm, early and late gastrulation. **a**–**d** The distribution and intensity of *DmFKBP12* mRNA along with longitudinal axis of *Drosophila* at syncytial blastoderm, cellular blastoderm, early gastrulae and late gastrulae. The stage indicating on the left for *Drosophila* development is quoted from Ref [35]. CN is short for Cleavage nucleus; YK, Yolk; BL, Blastoderm cell; ST, Stomodaeum; SLD :Salivary gland duct; MG, Midgut; PMG, Posterior midgut rudiment; FG, Foregut; MP, Malpighian tubules; VNS, Ventral nervous system; PMUS, Pharyngeal musculature; PH, Pharynx; PV, Proventriculus; HG, Hindgut; HY, Hypoderm; OES, Oesophagus

The above dynamic pattern on distribution of the *DmFKBP12* mRNA was observed within *Drosophila* embryogenesis within four stages. In syncytial blastoderm embryo, the mRNA signals were evenly distributed in both anterior pole and posterior pole (Figs. 6a–c, 7A). However, in cellular blastoderm embryo, the mRNA staining became un-even distribution with high density in anterior pole and less density in posterior pole (Figs. 6d–f, 7b). In early gastrulation stage, the un-even distribution became more noticeable by concentrating on neuronal tissue components in the zone of anterior pole, and less plus even expression of the *DmFKBP12* mRNA in the zone of posterior pole (Figs. 6g–i, 7c). In late gastrulation stage, the expression un-evenness and amount of the *DmFKBP12* mRNA converted to less or non in connective space and lumen cavity, and more in tissue-specific architecture (Figs. 6j–l, 7d). The un-even expression of the *DmFKBP12* mRNA in *Drosophila* embryo (Fig. 7a–d) immaculately supported the dynamic distribution of the DmFKBP12 protein presented the above data performed in four embryonic stages visualized with the patterns of protein density.

## Discussion

RyRs plus IP3R are mainly responsible for calcium sparks in skeletal muscle, cardiac myocytes, smooth muscle, neurons and other excitation cells [6, 7]. This calcium sparks can transform in different appearances or forms to perform their diverse functional molecular mechanism in cells of tissues where sparks locate. Ca^2+^ releasing via single tetramer RyR generating Ca^2+^ quark, via multiple adjacent complex producing Ca^2+^ wave and ER/SR origin Ca^2+^ local discretting Ca^2+^ puff play critical role in myocytes [6, 7]. In neuronal presynaptic terminal, RyR mediated Calcium releasing as Ca^2+^ syntilla, through Ca^2+^ synapse of a crossing-membrane structural and functional complex, are essential for neuronal transition [36, 37]. In vertebrate, three isoforms of RyR comprehend their biological compound commission with molecular mechanism in cells, in which regulations from FKBP12 and/or FKBP12.6 is required. In this study, our data obtained with dynamic localization of DmFKBP12 protein and *DmFKBP12* mRNA (Fig. 7), demonstrate functional essentiality of DmRyR-FKBP12 complex in embryonic development of *Drosophila melanogaster*.

It has been well recognized that FKBP12 and FKBP12.6 are critical regulators for Calcium-induced Calcium releasing through RyRs [6, 38, 39]. RyR1, RyR2 and RyR3 represent skeletal muscle specific isoform, cardiac isoform and brain specific isoform. Traditionally, FKBP12 is in charge of regulating skeletal muscle RyR1 Calcium channel, and FKBP12.6 interacts with cardiac muscle RyR2 by controlling EC coupling [38, 39]. Because of recent data demonstrated that FKBP12 functions in transgenic heart with its cardiac overexpression by generating lethal arrhythmia [19, 39–41], it is more reasonable that multifunctional regulation of these two proteins on skeletal or/and cardiac RyR could be much miscellaneous or complexed at tissue specific cellular microenvironment. Recently, FKBP12 was linked to IP3R by regulating calcium release function in cancer cells [42]. The data indicated that FKBP12 could play as multi-functional regulator working for both RyR and IP3R in charge of associating these two critical calcium channel proteins while cell carrying on the pathophysiological function. It is certain that DmRyR-FKBP complex contributes its important role within *Drosophila* development according to our result although no report make sure yet on embryonic function of IP3R in *Drosophila.*

**Fig. 8:**
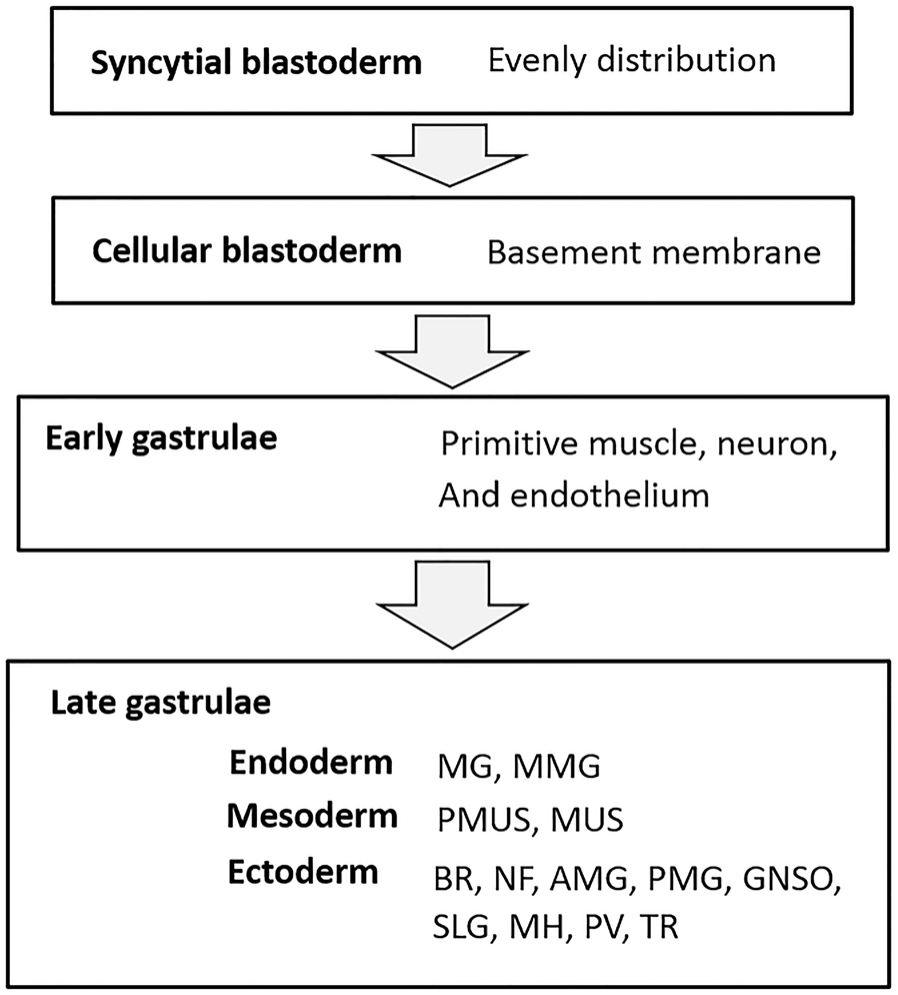
The Dynamic distribution of DmFKBP12/Calstabin protein in four development stage of *Drosophila melanogaster.* Full names of the developing tissues or organs were listed in Abbreviations by following standard terminology [45, 46]

*Drosophila* homologs of two mammalian intracellular CICR Ca^2+^ channels RyR and IP3R were first identified in the mesoderm of early stage-9 embryos, somatic precursor myocytes and neuronal tissues [42]. Then full-length of cDNA encoding DmRyR were cloned, and channel function of full-length and carboxyl-terminal with TMD channel portion were characterized systematically [16, 43, 44]. The critical function for larval development is characterized with hypomorphic allele RyR(16) mutation on muscle contraction and Ca^2+^ channel activity [21]. Although a present work consistent to approve the importance DmRyR, for instance, that oxidative stress induced stem cell proliferation in *Drosophila* midgut through activating TRPA1/RyR-mediated Ca^2+^ signaling, the DmFKBP12 as critical regulator of the Ca^2+^ signaling are perceptibly less investigated (Fig. 8).

The *Drosophila* homolog of mammalian RyRs has been investigated previously, and comprehensions on DmRyR are relatively well known such as that an only RyR gene was identified in insect instead of three isoforms in mammalian [16, 42–44]. However, very limited information documented from published data about insect RyR regulator DmFKBP12. The DmFKBP12 was identified on function of extension both health and life spans using loss of *DmFKBP12* gene function model in *Drosophila* muscle [20]. This work claimed that the absence of DmFKBP12 in the cytoplasmic of *DmFKBP12* mutant muscle dissociated RyR from response to oxidation and resulted in earlier aging. Our data of *DmFKBP12* cloning from earlier embryo (Fig. 1) extended that the function of DmFKBP12 perform its early contribution to fly embryo. The DmRyR-FKBP12 complex may already initiate their role not only in muscle but also in brain because DmRyR is only one RyR *Drosophila* homolog representative of mammalian three isoforms as we discussed above.

Molecular cloning of DmRyR cDNA provided the solid evidence of DmRyR expression in *Drosophila* lava [16]. As the crucial regulator of this CICR pathway along with binding protein of IP3R as well [47], in which four DmFKBP12 control opening of ER/SR DmRyR tetramer, the expression and function of DmFKBP12 should be carried out in the lava stage. Because of, as discussed above, RyR-mediated CICR exhibiting with calcium spark and calcium wave robusting from fertilization of many animals [48–51], it is judicious that the DmRyR-FKBP12 complex functions as early as the same stage. Our data in this investigation demonstrated that the DmFKBP12 distributed in subsurface cortex apposition to the plasma membrane in syncytial blastoderm (Figs. 1, 2, and 6a–c). We believe that the *Drosophila* DmRyR-FKBP12 as an ancient conserved complex performs its essential role throughout entire life of *Drosophila melanogaster*. Our data showed that the protein expression of *Drosophila* FKBP12 is significant different at four embryonic stages with the same mRNA level. This result strongly indicated that the existence of regulation pathway in the *Drosophila* embryonic development could link to the involvement of more proteins such as BAP1 and PTEN, which regulate by targeting on IP3R3-mediated Ca^2+^ flux to mitochondria in mammal cell [52–54].

Furthermore, our results suggested a proficient strategy that binding ligands such as Tacrolimus (FK506), sirolimus (rapamycin) and chemicals derived from them, have potential to utilize on developing pesticide against early development stage *Drosophila melanogaster* by targeting on the embryonic DmRyR-FKBP12 complex. When tacrolimus and sirolimus as immunosuppressant molecule bind on FKBP proteins including DmFKBP12, this binding would decline or even fail the formation of DmRyR-FKBP12 or DmIP3R-FKBP12. Therefore, the cellular calcium signaling pathway would be disrupted accompanying with death of muscular and neuronal tissues while the dysfunction of DmRyR FKBP12 occurs on *Drosophila* embryo in four stages in development.

## Conclusion

Calcium signaling plays critical roles in many biological functions with Ryanodine and IP3 Receptor mediated CICR mechanism at cell molecular level. Calcium spark along with its other appearances evidently performs function with RyR/IP3R-FKBP complex associated calcium signaling from fertilization, via skeletal, cardiac and smooth muscle contraction, to presynaptic terminal neuronal cells. Our data suggested that DmRyR-FKBP12 complex plays an essential role as the regulator of DmRyR through CICR pathway. As absence of FKBP12 directly causes early newborn lethality with cerebral edema and severe arrhythmic defect representative with conduction error of cardiomyocyte in vertebrate, our data approved the present of DmFKBP12 in *Drosophila* early embryonic development and investigated the dynamic distribution of DmFKBP12 protein. The DmFKBP12 subsurface distribution apposition to the plasma membrane and cortex distribution within the multinuclear plasma directly supports the critical role performed through DmRyR-FKBP12 complex in *Drosophila melanogaster* embryo as early as at the stages of syncytial blastoderm, via cellular blastoderm and to late gastrula. Our data propose a unique potential to develop insect specific and long-term (from early embryo and adult) effective pesticide by targeting on insect embryonic RyR-FKBP12 complex against insect CICR calcium signaling pathway.

## Additional file

**Additional file 1: Figure S1.** Sequence analysis of *Drosophila melanogaster* domain. The cDNA DmRyR domain cloned form *Drosophila* embryo as DmRyR domain (I + II) (A, C) and DmRyR domain (III + IV) (B, D). The sequences of DmRyR domain (I + II) and DmRyR domain (III + IV) correctly matched the document in NIH gene bank (NM_079068.5).

## Abbreviations

AMG: anterior midgut rudipighian
AP: anterior pole
BLD: blastoderm
BM: basement membrane
BR: brain supraesophageal ganglion
CaSR: calcium-sensing receptor
CF: cephalic furrow
CICR: calcium-induced calcium release
CN: cleavage nucleus
DIG: digoxigenin
DmFKBP: *Drosophila* FKBP
DmRyR: *Drosophila melanogaster* RyR
ER: endoplasmic reticulum
FKBP: FK506-binding protein
FG: foregut
GB: germ band
GNSO: suboesophageal ganglion
GP: glycoprotein
H&E: hematoxylin–eosin
HG: hindgut
HY: hypoderm
IP3R: inositol 1,4,5-trisphosphate receptor
ITG: integrins
NF: nerve fiber
MG: midgut
MH: mouth hook
MGC: midgut caecum
MMG: middle midgut
MP: malpighian tubules
MUC: head muscle
MUS: muscle
NBL: neuroblasts
NF: nerve fibers and tracts
OES: oesophagus
PASM: periodic acid-silvermetheramine
PH: pharynx
PMG: posterior midgut rudiment
PMUS: pharyngeal musculature
PP: posterior pole
PV: proventriculus
PVDF: polyvinylidene difluoride
RT-PCR: reverse transcription polymerase chain reaction
RyR: ryanodine receptors
SG: supraesophageal ganglion
SLG: salivary gland
SLD: salivary gland duct
ST: stomodaeum
SV: salivary gland
TR: trachea
VNS: ventral nervous system
YK: yolk
