# Supplementary material for: Dynamics expression of DmFKBP12/Calstabin during embryonic early development of Drosophila melanogaster
Source: Cell Biosci. 2019 Jan 8;9:8. doi: 10.1186/s13578-019-0270-6 (PMC6325743; doi:10.1186/s13578-019-0270-6)

Additional materials

Total RNA of the embryos was extracted with Trizol reagent (Invitrogen). Then reverse transcription polymerase chain reaction (RT-PCR) (Life Technologies) used to amplify first-strand of DmRyR domain of the 1st strain cDNA from 5 µg of purified total RNA with phenol:chloroform:isopropanol (25:24:1). Forward primer-1 (Primer-F1, 5'- agatgtgggctctaaaca -3') and reverse primer-1 (Primer-B1, 5'- tgaagatctcgttgggca -3'), forward primer-2 (Primer-F2, 5'- gagacatccgatccgata -3') and reverse primer-2 (Primer-B2, 5'- cctcgttctggaattcgt -3') were designed according to the published cDNA sequence of DmRyR obtained Drosophila melanogaster. The plasmids of cDNA DmRyR domain were purified with phenol:chloroform:isopropanol (25:24:1). The sequences of DmRyR domain (I+II) and DmRyR domain (III+IV) were inspected for confirmation on their correctness provided in Supplementary Figure S1A, B, C and D.

**Figure S1 Sequence analysis of *Drosophila melanogaster* domain** The cDNA DmRyR domain cloned form *Drosophila* embryo as DmRyR domain (I+II) (Figure S1 A and C) and DmRyR domain (III+IV) (Figure S1 B and D). The sequences of DmRyR domain (I+II) and DmRyR domain (III+IV) correctly matched the document in NIH gene bank (NM_079068.5).


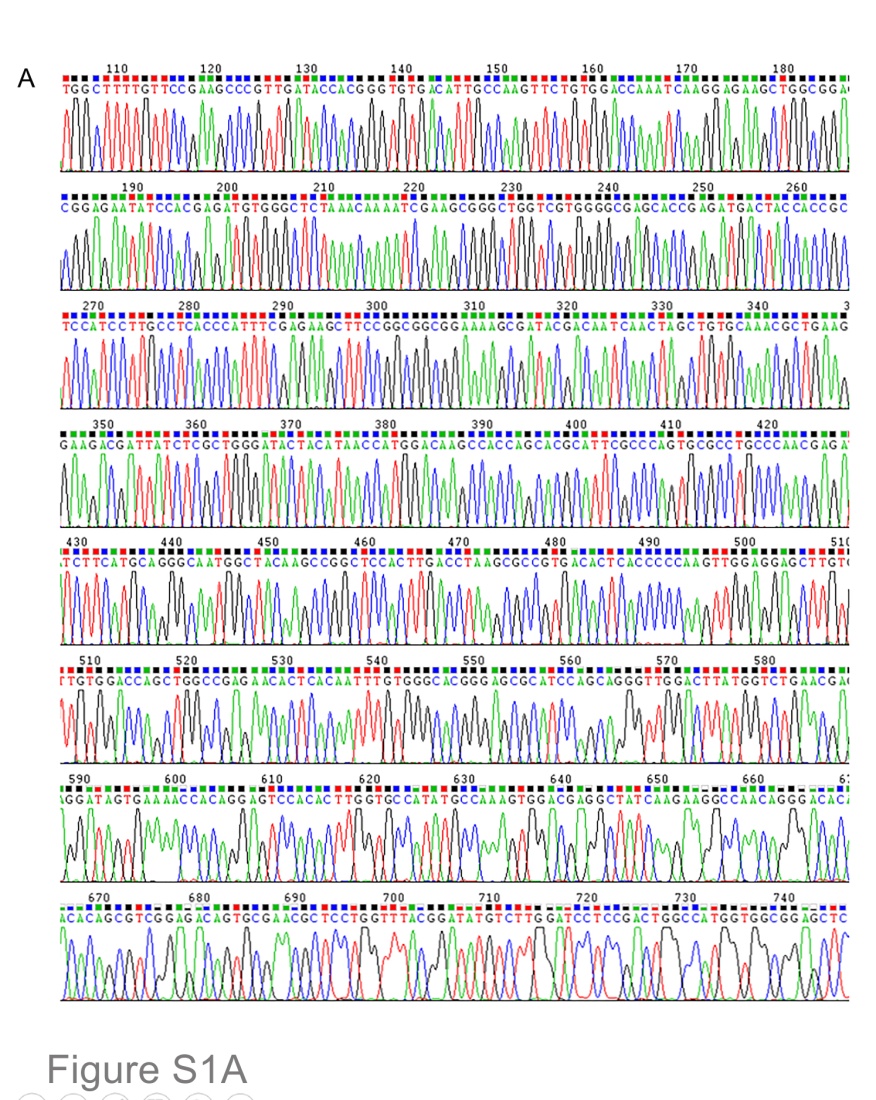


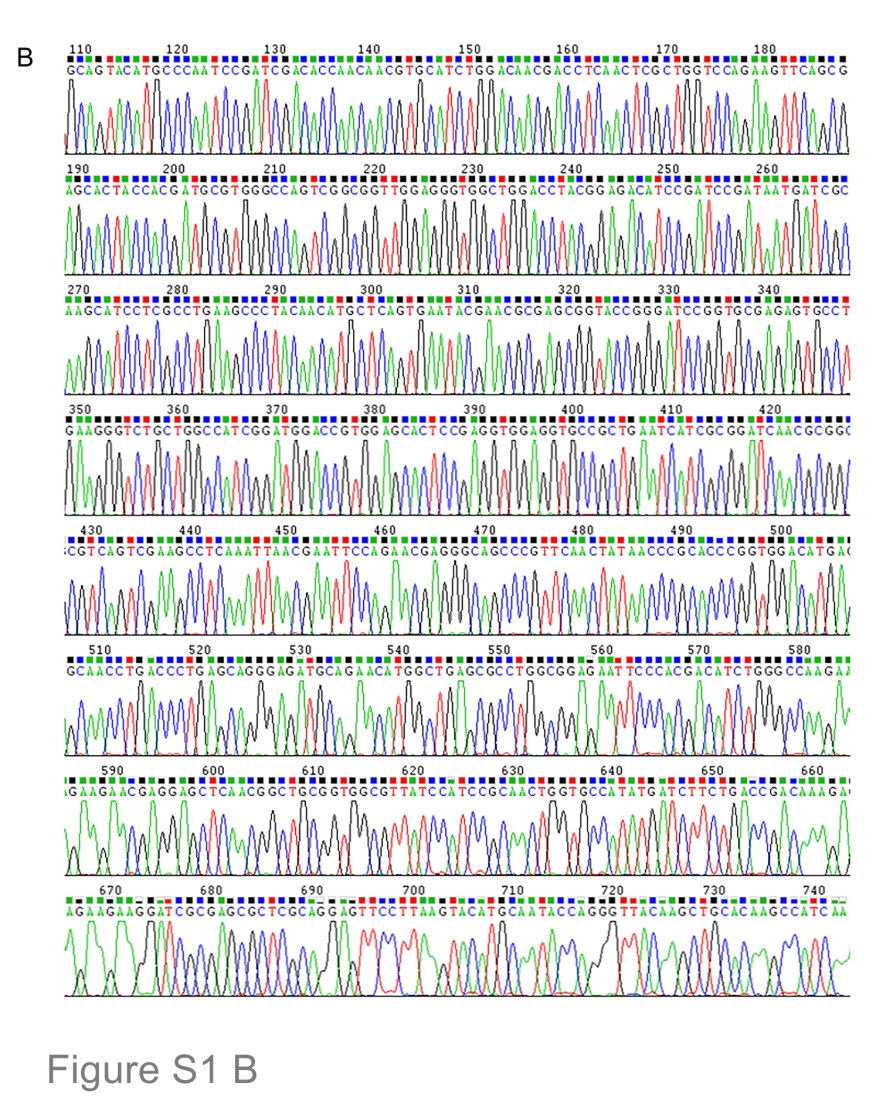


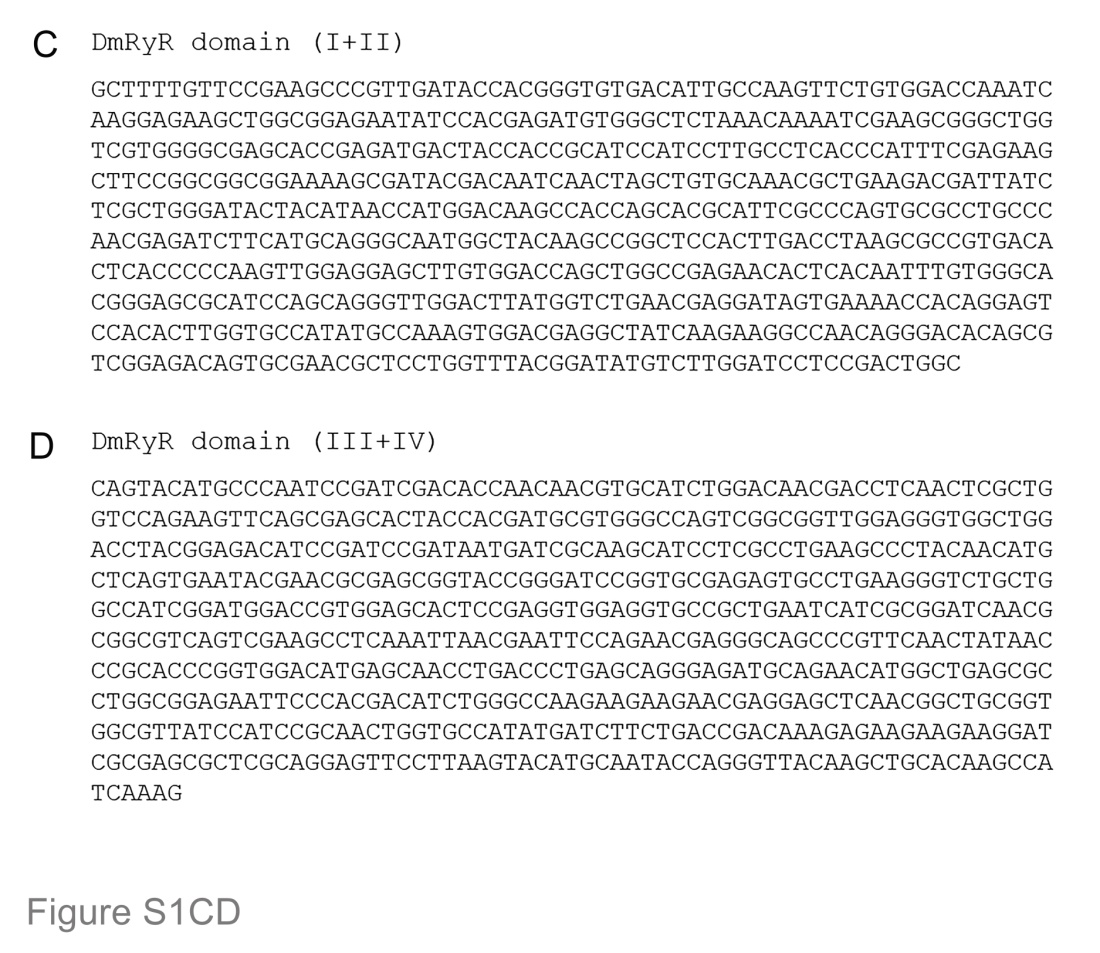

Supplement: Supplementary file 1 — Additional file 1: Figure S1. Sequence analysis of Drosophila melanogaster domain. The cDNA DmRyR domain cloned form Drosophila embryo as DmRyR domain (I + II) (A, C) and DmRyR domain (III + IV) (B, D). The sequences of DmRyR domain (I + II) and DmRyR domain (III + IV) correctly matched the document in NIH gene bank (NM_079068.5). [file 13578_2019_270_MOESM1_ESM.docx]
